# Autoantibodies as Diagnostic Markers and Mediator of Joint Inflammation in Arthritis

**DOI:** 10.1155/2019/6363086

**Published:** 2019-10-27

**Authors:** Qinghua Fang, Jiaxin Ou, Kutty Selva Nandakumar

**Affiliations:** School of Pharmaceutical Sciences, Southern Medical University, Guangzhou, China

## Abstract

Rheumatoid arthritis is a systemic, polygenic, and multifactorial syndrome characterized by erosive polyarthritis, damage to joint architecture, and presence of autoantibodies against several self-structures in the serum and synovial fluid. These autoantibodies (anticitrullinated protein/peptide antibodies (ACPAs), rheumatoid factors (RF), anticollagen type II antibodies, antiglucose-6 phosphate isomerase antibodies, anticarbamylated protein antibodies, and antiacetylated protein antibodies) have different characteristics, diagnostic/prognostic value, and pathological significance in RA patients. Some of these antibodies are present in the patients' serum several years before the onset of clinical disease. Various genetic and environmental factors are associated with autoantibody production against different autoantigenic targets. Both the activating and inhibitory Fc*γ*Rs and the activation of different complement cascades contribute to the downstream effector functions in the antibody-mediated disease pathology. Interplay between several molecules (cytokines, chemokines, proteases, and inflammatory mediators) culminates in causing damage to the articular cartilage and bones. In addition, autoantibodies are proven to be useful disease markers for RA, and different diagnostic tools are being developed for early diagnosis of the clinical disease. Recently, a direct link was proposed between the presence of autoantibodies and bone erosion as well as in the induction of pain. In this review, the diagnostic value of autoantibodies, their synthesis and function as a mediator of joint inflammation, and the significance of IgG-Fc glycosylation are discussed.

## 1. Introduction

Rheumatoid arthritis (RA) is one of the most common autoimmune diseases, which affects approximately 1% of the world population, and is characterized by autoantibody production, synovial inflammation, cartilage destruction, and bone erosion [1]. RA occurs when the immune system mistakenly attacks our own body's tissues, causing systemic inflammation damaging not only the articular joints but also a wide variety of other organs including the skin, eyes, lungs, heart, and blood vessels. Many serological studies have shown that a great diversity of well-characterized autoantigens exists in RA patients, for example, citrullinated proteins and peptides, including fibrin; components of articular cartilage (collagen type II, CII); circulating serum proteins including antibodies and acute phase proteins; nuclear components; enzymes (calpain inhibitor protein); and other target antigens [2]. In RA, an increased number of autoantibodies directed against these self-antigens such as rheumatoid factors (RF) and anticitrullinated protein antibody (ACPA) are commonly prevalent.

The inflammation in RA causes joint damage at the early stages itself leading to bone erosion and functional disability. RA patients often have immune system dysfunction and are associated with extra-articular manifestations involving several organs [3]. With the continuous development of medical standards, the progress of RA patients can be alleviated by regular treatment but it cannot be completely cured. Therefore, exploring the pathogenesis of RA is very important for developing precise treatments and new drug targets. Although being a considerable health problem, information about the disease pathways and etiology is far from clear [4] because of the heterogeneity of the disease phenotype. A large number of studies have found that abnormally increased immune cells (T cells, B cells, macrophages, and neutrophils) and immune molecules (cytokines, autoantibodies, and heat shock proteins) are present in the synovial tissue and fluid of RA patients, which suggest that the release or activation of them may be involved in the initiation and perpetuation of RA. Therefore, extensive and in-depth understanding of these factors and their interactions in the development of RA is of great significance for its prevention and treatment.

In the 1940s, presence of RF in the serum of RA patients was identified and consequently used as the “gold standard” for the diagnosis [5]. Early diagnosis and treatment can often delay and prevent joint deformities, improving the quality and duration of life for the patients, so it is a prerequisite to identify the patients as early as possible. However, if RA is diagnosed according to the current diagnostic criteria of the American College of Rheumatology (ACR), it is usually not early. The discovery of new specific autoantibodies to improve the early diagnosis rate has become a hot topic in current research. More than ten autoantibodies related to RA were identified, which have greatly helped to develop new early diagnosis and prognosis methods. In recent years, the identification of anticitrullinated protein antibodies as a new diagnostic marker for RA is a new milestone in this field.

Detection of autoantibodies is commonly used to confirm clinical diagnosis or to help define a subset of patients in the diagnostic category [2]. In this review, how specific autoantibody responses change and evolve over time to become more pathogenic, interactions between different autoantibody types, their synthesis, and the role of Fc glycosylation will be discussed. The implications of these findings for the clinical practice are briefly discussed.

## 2. Autoantibodies in RA

It has been recognized for some time now that in the natural history of RA, there is a phase for developing autoimmunity that precedes the onset of clinical symptoms in a large proportion of patients. The most prominent players in this preclinical phase are the autoantibodies, and although no definitive causal link with the development of arthritis has been established, autoantibodies have been shown to induce arthritis in different mouse models [9–11].

At present, autoantibodies related to RA include ACPA, antikeratin antibodies (AKA), antiperinuclear factor (APF), antifibronectin antibody (AFA), antimutated citrullinated vimentin (anti-MCV) antibody, anti-Sa antibody, RF, antiglucose-6 phosphate isomerase (anti-GPI) antibody, anticarbamylated protein (anti-CarP) antibody, antiacetylated protein antibody, antinuclear antibody (ANA), antiheterogeneous nuclear ribonucleoprotein (anti-hnRNP/RA33) antibody, anti-Bip antibody and anticalcitostatin antibody (ACAST), and anti-CII antibody [12, 13]. Presence of these antibodies in RA is of great significance for early diagnosis and treatment. To prevent irreversible joint damage, early diagnosis and treatment initiation within the first three months of disease onset is essential [14]. Apart from diagnostic value, autoantibodies such as ACPA, RF, and other antimodified protein antibodies are considered as important risk factors for the development of RA and probably play an important role in its pathogenesis.

While the presence of autoantibodies is an important risk factor for future RA and part of the ACR/EULAR RA classification criteria, it does not always lead to the development of disease [15]. This may be explained by the heterogeneous character of the various autoantibody responses present in the individuals being at risk for RA development with different intrinsic properties such as affinity, specificity, isotype composition, and glycosylation. These properties translate into different capabilities for modulating inflammation. Furthermore, autoantibody responses can evolve their pathogenic properties in the period leading up to and during the clinical manifestations of autoimmunity.

## 3. ACPA

**Figure 1:**
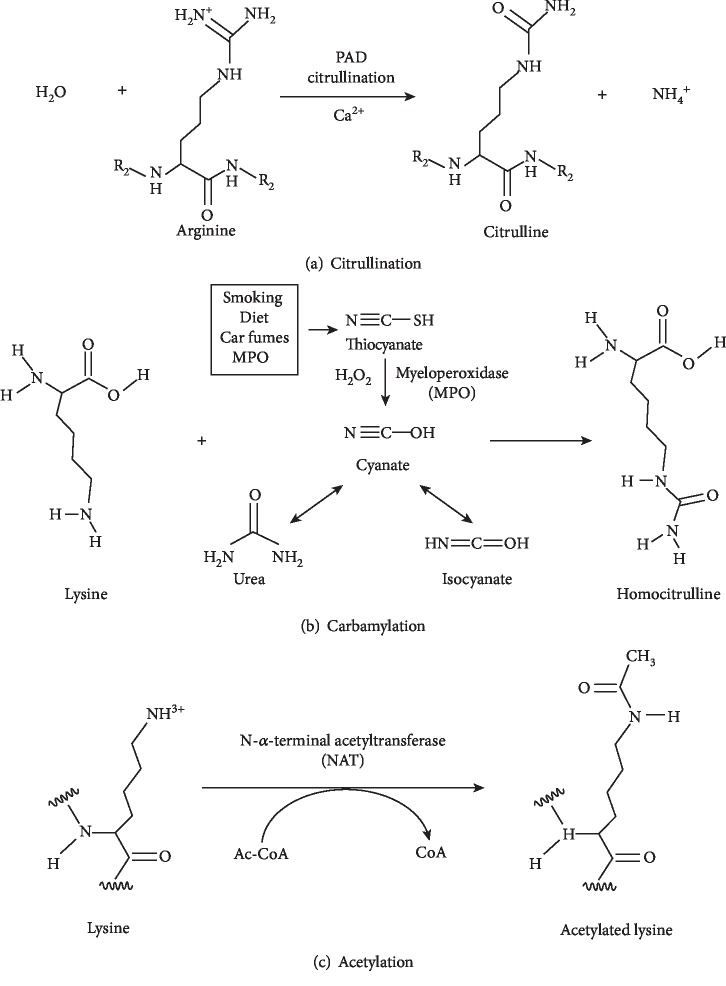
Schematic overview of the posttranslational modifications citrullination, carbamylation, and acetylation. The process of citrullination modifies an arginine (R) present in the amino acid sequence of a protein into citrulline, whereas the process of carbamylation modifies a lysine (K) into homocitrulline. Citrullinated and carbamylated proteins are recognized by anticitrullinated protein antibodies and anticarbamylated protein antibodies, respectively. The process of citrullination is mediated by peptidyl arginine deiminase (PAD) enzymes, whereas the process of carbamylation is a chemical reaction driven by cyanate. Acetylation is catalyzed by acetyl transferases. Ac-CoA: acetyl-CoA; H_2_O_2_: hydrogen peroxide; MPO: myeloperoxidase [6–8].

APF [16] was confirmed to be a specific antibody to RA in 1964 and found to be present early in the disease. Since then, AKA [17], AFA [18], and anti-Sa antibody [19] were identified as highly specific markers for the diagnosis of RA. All these antibody targets are chemically related, their epitopes having citrullinated structures resulting from the posttranslational modification of arginine, and hence are called ACPA, which are specifically present in RA patients [20]. The enzyme peptidyl arginine deiminase (PAD) catalyzes the modification of arginine residue of a protein into a citrulline residue by an enzymatic reaction called citrullination (Figure 1(a)). The citrullinated proteins are often found in the joints of RA patients but are not specific for the disease. In general, the citrullination of the protein may alter its tertiary structure, interaction with other molecules, cleavage regions, and its solubility to enhance its immunogenicity, which could stimulate the immune response to produce corresponding antibodies [21, 22]. It has been found that a large number of abnormally proliferating macrophages and lymphocytes infiltrate into the synovial tissue of RA patients. PAD enzymes, especially PADI4, are activated in these cells, leading to protein citrullination in the synovial membrane, which in turn stimulates B cells to produce large amounts of ACPA, resulting in ACPA immune complexes (IC). The interaction and deposition of IC can induce the production of a variety of cytokines, causing chronic persistent inflammation of the synovium [23]. Synovial tissue protein citrullination has been shown to play an important role in the disruption of immune tolerance mechanisms in RA.

Under normal circumstances, citrullinated proteins degrade regularly and do not trigger any related humoral immune response, so the presence of citrulline proteins itself does not necessarily lead to chronic inflammation [24]. Also, it is a process that exists in a wide range of inflammatory tissues, indicating that it is an inflammatory phenomenon that should usually be tolerated by the immune system. Citrullination seems to be related to many of these accessory physiological processes, such as the pathway of cell death, in which intracellular calcium concentrations rise to higher levels than physiological conditions. Therefore, PAD is activated during apoptosis, autophagy, and NET formation, and it is well known that these processes are related to autoimmunity. Hence, citrullination may be considered as an inflammatory dependent process that plays a central role in autoimmune diseases [25].

In the past decade, ACPAs have emerged as suspects in the development and/or progression of RA. The abnormally expressed citrullinated protein levels in the RA inflammatory synovial membrane are directly related to the severity of the disease. Intriguingly, ACPA can also be found in individuals before the onset of clinical symptoms. In these instances, the ACPA response seems to be in its infancy, recognizing only a few citrullinated antigens and not using the full isotype repertoire. These characteristics of the ACPA response mature before the precipitation of the clinical disease.

The targets of ACPA include both endogenous autoantigens (the vast majority) and exogenous antigens (a few). According to the data so far available, most of these antigens are expressed in organs and tissues involved in the immunopathology of RA. This group of deiminated autoantigens includes structural constituents of the joints (CII), proteins that form deposits in inflamed joints (fibrinogen/fibrin), cytoplasmic proteins (immunoglobulin binding protein (BiP) and vimentin) that are highly expressed and citrullinated in the inflamed synovia, and nuclear proteins (histones) that become accessible to the immune system under inflammatory conditions in the neutrophil extracellular traps (NET). Thus, several citrullinated proteins have been described as targets of ACPA: filaggrin [26], vimentin [27, 28], *α*-enolase [29], and other proteins but more particularly the *α*- and *β*-chains of fibrin [30]. Citrullinated fibrinogen, CII, and vimentin, which are known as natural antigens and components of immune complexes, are expressed abundantly in the inflammatory tissues of RA patients and may play important roles in the pathological process of RA.

There is considerable variability of the estimated prevalence of autoantibodies in RA among various studies conducted so far (Tables 1 and 2). First, a lower prevalence of RA was reported in rural compared to the urban regions where it appears to be close to that of Caucasian populations, suggesting an environmental influence related to western lifestyle and/or industrialization [31, 32]. Indeed, in Caucasian cohorts, besides environmental factors, several genetic factors have been identified that predispose to the disease, especially the major susceptibility loci being the HLA-DRB1 alleles [33]. These alleles code for proteins that contain similar amino acid sequences (QKRAA, QRRAA, or RRRAA), also known as “shared epitope” (SE), and are present in 60–80% of Caucasian RA patients [34]. In Black African patients, the frequency of the SE alleles was also found to be higher in RA patients than in controls, for example, 40% vs. 10%, in a cohort from South Africa and 30% vs. 10% in a Cameroonian cohort, but it was always in lower frequency than the Caucasian patients (70% vs. 30%, for RA and controls, respectively) [35]. Interestingly, in Caucasian patients, the SE alleles are predominantly associated with ACPA-positive RA. Tables 1 and 2 show that patients tested in different countries have varied responses to different antigens, which might explain the differences observed in the incidence of RA; however, citrullinated peptide is not a natural antigen [36]. Therefore, identifying the natural antigen targeted by anti-CCP *in vivo* is of great significance for designing the early diagnosis tests for RA.

ACPAs can induce damage when activating classical and alternative complement pathways. It is also capable of triggering immune cell responses via Fc receptors (Fc*γ*Rs). In this context, immune complexes containing ACPA and citrullinated fibrinogen have been shown to induce TNF-*α* secretion on macrophages via binding to Fc*γ*Rs [4]. ACPAs can also bind osteoclast progenitor cells and directly promote them to differentiate into bone-resorbing osteoclasts. Interestingly, it was observed that bone loss begins even before the onset of clinical disease in ACPA-positive individuals, which suggests that these antibodies might play an independent role in initiating the bone damage [37]. Another mechanism for the ACPA-mediated proinflammatory effect may be through the formation of NET. Neutrophils release NETs containing chromatin associated with granules, which are not only capable of killing extracellular microorganisms but can also act as the source of autoantigens [38]. The role of NETs in producing citrullinated proteins is well recognized and was reported to be essential to generate ACPA [39]. In the form of immune complexes, ACPAs can also upregulate the production of proinflammatory cytokines. For example, combination of IgM-RF and ACPA promotes the production of proinflammatory cytokines *in vitro* [40].

## 4. Rheumatoid Factor

RF has been widely used in the diagnosis of RA since its discovery in 1940 as an antibody directed against serum gamma-globulin, which promoted the agglutination of sheep red blood cells sensitized by subagglutinating doses of rabbit antibodies [41]. RF is an autoantibody reacting against the Fc portion of IgG antibodies, produced locally by B cells present in the lymphoid follicles [42] and detectable in the serum of about 70% of patients with established RA but less frequently in early RA.

IgM-RF is the earliest discovered RA-related antibody and may be present many years before the onset of clinical disease; thus, its presence may also indicate an increased risk of disease development [43]. Current studies have shown that in addition to IgM-RF, multiple subtypes of RF can be detected in the serum of RA patients by ELISA, such as IgG, IgA, IgE, and IgD, which may predate disease onset by years [41]. Currently, IgM-RF is listed as the only serological indicator in the diagnostic classification of RA and is one of the most widely used biomarkers [44]. The RF-positive rate in RA patients is about 60% to 80%, but the rate is lower (50-60%) during early stages [45]. However, there is also a certain positive rate in patients with other systemic autoimmune diseases such as Sjogren's syndrome, mixed cryoglobulinemia, systemic lupus erythematosus (SLE), mixed connective tissue disease (MCTD), and primary biliary cirrhosis and in infectious diseases like chronic tuberculosis, hepatitis C, Epstein-Barr virus infection, cytomegalovirus infection, and subacute bacterial endocarditis [41, 46, 47]. Even in healthy people, RF levels increase with age, and positive reactions can be seen in 5% of young people and up to 25% in the elderly [48]. High titers of RF, anti-CCP antibodies, or both are considered as serological hallmarks of RA [49]. Therefore, specificity of IgM-RF alone for RA diagnosis is considered to be insufficient. Similarly, detection of RF does not generally help in monitoring the disease, although it may help with the use of certain biologics, such as etanercept and infliximab, when levels of RF may decrease along with the clinical disease activity [50, 51].

Combining different isotypes is more specific than a single antibody. IgM, IgA, and IgG-RF are present in up to 52% of RA patients but also in fewer than 5% of patients with other rheumatic diseases. A number of studies have shown that the positive rate of IgG-RF in RA patients is 41.5% to 66% [52]. Moreover, IgG-RF has a higher specificity (91%) in the diagnosis of RA and correlates highly with the joint damage [53, 54]. Therefore, the combined detection of IgG-RF and other RA-related antibodies is of great significance for the diagnosis of RA. The combined occurrence of IgM- and IgA-RF has high diagnostic specificity for RA, but the presence of IgA and IgG-RF isotypes in the absence of IgM-RF is less specific, since they are also prevalent in patients with diverse rheumatic diseases [45]. The physiological role of RF under normal conditions includes promoting the stability of IgG bound to solid surfaces such as bacterial cell walls, enhancing immune complex clearance by increasing its stability and size, helping B cell uptake of immune complexes, thereby efficiently presenting antigens to T cells, and facilitating complement fixation by binding to IgG containing immune complexes [45].

The RF in RA patients is relatively of high affinity in nature, which is different from the poly-reactive and low affinity RF present in the healthy individuals [55]. Studies have shown that RF is a pathogenic autoantibody that plays a key role in the pathophysiology of RA [56]. In normal conditions, transient production of low-affinity IgM-RF is regularly induced by immune complexes and polyclonal B cell activators, such as bacterial lipopolysaccharide and Epstein-Barr virus [57]. The main role proposed for RF in RA is to form immune complexes, fix complement, and release chemokines, such as C5a, thus recruiting inflammatory cells (neutrophils) into the joints. Then, the activated inflammatory cells phagocytize the immune complexes and release proteolytic enzymes, causing joint tissue destruction. In addition, RF could also be involved in the retention of antigens within the joint. In this way, formation of immune complexes at the sites of synovial inflammation will be induced, and complement and leukocyte infiltrations will be ensured [58]. RF-specific B cells migrate into the synovium of RA patients, expressing multiple antigens to T cells, which may contribute to the continuation of local inflammatory responses and the expansion of synovial RF products. Therefore, RF may prolong the survival of B cells and thus maintain their own production.

Unlike lgM-RF, aggregated IgA-RF activates the complement through the alternative pathway to participate in the pathological processes. Polymerization of IgA-RF and IgG into an immune complex can stimulate neutrophil release of elastase, cathepsin, lysozyme, and myeloperoxidase to participate in the bone destruction [59]. Nonpolymeric IgA-RF can also regulate the phagocytosis of monocytes. It was observed that IgA-RF can selectively activate macrophages to produce prostaglandins, IL-1, TNF-*α*, and other cytokines causing degradation of the bone matrix and damage to the cartilage. Interestingly, memory B cells expressing the IgA-RF receptor FcRL4 were also found in the joints of RA patients, which via RANKL expression can contribute to joint destruction [60].

RF plays a pivotal role in the differential diagnosis and prognosis of RA patients [57]. It has been shown that RF is useful in predicting the development of RA, as the detection of IgM-RF can be used as a marker of inflammatory activity [61]. The preclinical appearance of RF isotypes in the serum follows a specific sequential evolution: first IgM, then IgA, and finally IgG-RF [62]. These three autoantibodies have different meanings in clinical diagnosis and should be treated differently: Elevated levels of IgM- or IgA-RF alone suggest the possibility of infection. If the IgM- or IgA-RF titer is progressive even after anti-infective treatment, the possibility for positivity for RA is high. On the other hand, elevated IgG-RF alone suggests a higher probability of RA, and if the antibody titer is significantly increased, it may be associated with vasculitis. If IgM- and IgA-RF increase simultaneously, then the possibility for RA is high. If all the RF isotypes (IgM, IgG, and IgA) increase simultaneously, RA can be diagnosed positively but it still needs to be differentiated from other diffuse connective tissue diseases.

High titers of RF have been associated with worse prognosis, more aggressive articular disease, increased disease activity, reduced rates of remission, higher prevalence of extra-articular manifestations, and increased morbidity and mortality, especially when present in combination with ACPA [62, 63]. In addition, when the patient's serum has high levels of IgM- and IgA-RF, the disease progresses rapidly suggesting that bone erosion and bone destruction are prone to occur and the continuous increase of IgM- and IgG-RF in the serum can lead to poor prognosis [64]. However, studies showing the clinical usefulness of RF in monitoring disease activity and treatment response are limited. We still need more indicators to judge and understand various conditions or subtypes of RA. As a sensitive marker of acute phase proteins and inflammatory responses *in vivo*, C-reactive protein (CRP) can effectively compensate for the negative results of partial RF screening in the diagnosis of autoimmune diseases, which could effectively avoid the occurrence of misdiagnosis and missed diagnosis [65, 66].

## 5. Antiglucose-6 Phosphate Isomerase (Anti-GPI) Antibodies

GPI is an important enzyme in glycolysis and gluconeogenesis in the body and can be secreted outside the cell as a cytokine or growth factor. However, GPI is also an autoantigen in RA. In a T cell receptor transgenic (KBN) mouse model, continuous production of GPI-specific antibodies was detected [67]. Transfer of these antibodies to healthy mice induced arthritis [68]. Complement components [69], especially the alternative pathway of activation [70], cells bearing Fc*γ*Rs [71], and various inflammatory cytokines, play an important role in the disease pathogenesis. Immunization of mice with recombinant GPI [72] or GPI peptides [73, 74] can also induce arthritis in naive mice. In 2001, Schubert first reported that anti-GPI antibodies were associated with RA [75]. Subsequently, Jouen et al. reported the positive rate of anti-GPI antibody in the serum of RA patients as about 45.4%, but the specificity was only 75.0% and the appearance of antibodies was not correlated to the prognosis of RA patients [76]. Moreover, anti-GPI antibodies were found to be not specific for RA [77].

## 6. Anticarbamylated Protein Antibodies

Anticarbamylated protein (anti-CarP) antibodies are a new type of autoantibodies described in RA recently. Similar to citrullination, carbamylation is a kind of posttranslational modification of proteins, which provides a source of new epitopes that can be recognized as non-self-antigens [78]. Carbamylation is a chemical reaction mediated by cyanide in which a lysine is converted into a homocitrulline (Figure 1(b)). Certain conditions, for example, renal disease, smoking, and inflammation, can increase cyanide levels and thus carbamylation. Presence of these modified sequences of amino acids may provoke specific autoantibody production in RA. Antibodies in the serum of RA patients can discriminate citrullinated and carbamylated antigens. Therefore, this antibody system is independent of ACPA [57]. However, cross-reactivity between ACPA and anti-CarP antibodies is observed. Nevertheless, at least a subpopulation of anti-CarP seems to be independent of ACPA and associated with erosive disease [79].

In an animal model, it was shown that carbamylated proteins can trigger primary immune responses inducing chemotaxis, T cell activation, antibody synthesis, and production of IFN-*γ*, IL-10, and IL-17. The activation of T cells and a strong antibody response enabled the recognition of carbamylated and citrullinated peptides within the joints, which further contributed to the development of erosive arthritis [79]. Carbamylated and citrullinated peptides complement each other in the generation of the autoimmune responses. The immune-activating effects of carbamylation enhance the arthritogenic properties of citrullinated peptides, thus providing a novel mechanism for the pathogenesis of autoimmune arthritis [80]. Verheul and colleagues have identified carbamylated alpha 1 antitrypsin (A1AT) as an antigenic target of anti-CarP antibodies in RA patients [81].

Similar to the citrullination, increased carbamylation alone does not seem to be sufficient to break the tolerance to induce autoimmunity. Only 12% of patients with renal disease harbor anti-CarP antibodies compared to approximately 44% of RA patients [82]. Anti-CarP antibodies consist of 45% IgG and 43% IgA isotypes. Notably, anti-CarP antibodies may occur in 16% to 30% of ACPA-negative patients (16% IgG and 30% IgA isotypes) [83]. Moreover, anti-CarP antibody was reported to be associated with radiographic progression in patients negative for RF and ACPA. However, diagnostic classification of RA patients did not improve by adding anti-CarP testing, as RF and ACPA are already good predictors for the disease [83]. Overall, the sensitivity of anti-CarP is lower than ACPA; however, the simultaneous assessment of anti-CarP and ACPA may be very beneficial to identify RA patients [84].

## 7. Antiacetylated Protein Antibodies

The latest addition to antimodified protein antibodies (AMPAs) in RA patients is antiacetylated protein antibodies present in approximately 40% of RA patients, mainly in the ACPA-positive group. Similar to citrullination, acetylation may be involved in the pathogenesis of RA by triggering the production of autoantibodies and/or by producing antibody response targets in rheumatoid joints [85]. Acetylated lysine resembles homocitrulline, but the side chain terminal amine is replaced by a methyl moiety in acetylated lysine (Figure 1(c)). Acetylation is a reversible enzymatic process in which acetyl groups are added to free amines of lysine residues by lysine acetyltransferases (KAT) [86]. Protein lysine acetylation is a key posttranslational modification in cellular regulation, especially in histones and nuclear transcription regulators. Recently, mice carrying a HDAC1 deletion in their CD4^+^ T cells are reported to be protected from autoimmune disease [87]. Moreover, acetylation of cytoplasmic proteins regulates metabolic pathways and enzymatic functions.

IgG and IgA antibodies against acetylated vimentin peptides were detected in 35% of patients with early arthritis. However, data showed that antiacetylated vimentin antibodies are relatively poor for predicting the development of anti-ACPA-negative RA. Their presence and frequency in established RA and their role in predicting disease severity and other clinically relevant outcomes in RA patients remain to be established. Detection rates in sero-negative RA patients were comparable to patients with resolving arthritis rendering it unlikely that these antibodies will be a new biomarker helpful for diagnosing RA [85]. However, antiacetylated protein antibodies might provide useful new insights into pathophysiology, especially in the era in which the microbiome seems to become increasingly important. Acetylation is an enzymatic process, which can be affected by bacteria, although the underlying mechanism is unclear. Therefore, antiacetylated antibodies could provide a possible new link between microbiome dysbiosis and the development of autoimmunity in RA [88].

The diagnosis of RA usually depends on imaging examination, clinical characteristics, and results of autoantibody tests. However, the common clinical manifestations are not specific to RA, and the diagnostic values of autoantibodies are not considered as satisfactory [41, 150]. Therefore, it is necessary to establish alternate methods or discover new antibodies to further improve precise diagnosis. Fibrinogen is a precursor form of fibrin which deposits abundantly in the joints of RA patients. After the discovery of citrullinated fibrin in RA patients, research regarding the association between anticitrullinated fibrinogen (ACF) antibody and RA has gradually increased. One meta-analysis reported moderate diagnostic value for ACF in RA with a high specificity but limited sensitivity [151]. The sensitivity and specificity of ACF were similar to anti-CCP antibodies, so it may possess the similar diagnostic value in RA patients as anti-CCP antibodies [152]. However, the sensitivity of ACF is higher than that of IgM-RF and it is also related to the imaging progress of RA [153]. Therefore, ACF may contribute to the diagnosis of RA when combined with other antibodies and also in the clinical manifestations.

## 8. Natural and Pathogenic Autoantibodies

Even in the absence of an external antigen stimulation, natural autoantibodies (NAbs) can be secreted by B1a cells, which can present antigens efficiently, serving housekeeping functions and maintaining the homeostasis of the whole immune system. NAbs are not only limited to protecting the host from exogenous pathogens but can also act as key guard of the immune system by removing autoantigens and scavenging own tissues, such as dead or apoptotic cellular debris [154]. NAbs are mostly antibodies of the IgM isotype (also contain IgG and IgA isotypes) characterized by poly-reactivity, with low titer and low-to-moderate antigen-binding affinities [155]. Natural antibodies are germline- or close to germline-encoded variable regions directed against both microbial and altered self-antigens [156]. Interestingly, B1 cells also have an important role in the production of pathogenic autoantibodies in several autoimmune diseases, including RA.

**Figure 2:**
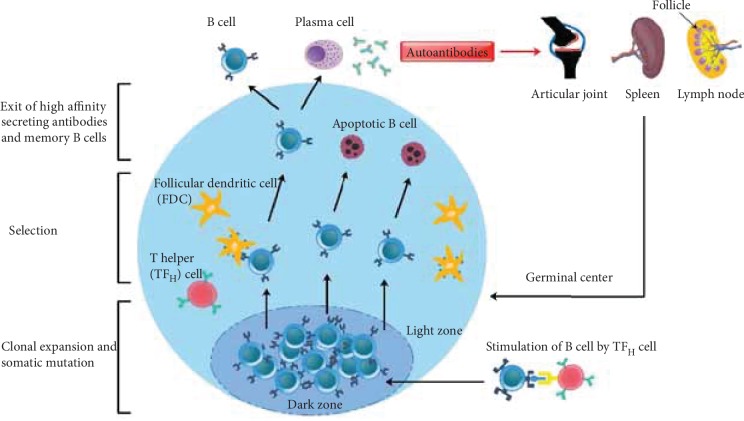
Germinal centers refer to sites within secondary lymphoid organs, lymph nodes, and the spleen where mature B cells proliferate, differentiate, and mutate their antibody genes by highly mutating somatic B cells to generate higher affinity (through somatic hypermutation) and class-switched antibodies. These cells developed dynamically after T-dependent antigen-activated follicular B cells. Formation of ectopic lymphoid aggregates with GC-like structures in the inflammatory tissues of RA patients is considered to contribute to the pathogenesis of arthritis.

High affinity autoantibodies having various effector functions, which are achieved in the germinal centers (GCs) of the secondary lymphoid organs, are essential for driving the autoimmune diseases. Although B cells can differentiate into short-lived plasma cells outside GCs, plasma cells matured within GCs produce more high affinity antibodies [157]. Follicular dendritic cell (FDC) networks play a pivotal role in maintaining GCs, as GC formation is abrogated in the absence of such FDC networks. Studies using gene-targeted mice have highlighted the nonredundant role of the inducible costimulatory molecule (ICOS), CD40, and lymphotoxin in the initiation and maintenance of GC niches [158]. In GC, follicular T helper (TF_H_) cells and B cells cooperate to mediate Ig class switching, affinity selection, generation of memory B cells, and antibody secreting plasma cells [159] (Figure 2). Various signaling molecules (for example, ICOS, CD40-CD40L, and signaling lymphocyte activation molecule- (SLAM-) associated protein (SAP)) are reported to be involved in TF_H_ cell-B cell interactions in the GCs. In the absence of help from TF_H_ cells, GC reactions were reported to be disrupted [160]. B cells present in the GC that are stimulated by antigen and TF_H_ cells differentiate into memory B cells and long-lived plasma cells secreting such high affinity antibodies. Many of the autoantibodies show characteristics of GC origin, suggesting defective selection of GC B cells in autoimmune diseases [161]. A specific role for TF_H_ cell-B cell interactions in the development of autoimmunity has been identified by studies done with *Roquin*^san/san^ mice. These mice are homozygous for a knockout in the Roquin (Rc3h1) allele, encoding a member of the RING-type ubiquitin ligase protein family responsible for RNA translation and stability in CD4^+^ T cells [162]. *Roquin*^san/san^ mice show spontaneous GC formation with an increased synthesis of pathogenic autoantibodies, which could be due to a defect in the selection process for autoreactive B cells in the GCs [162].

RA is a chronic inflammatory disease, and autoantibody-mediated pathology contributes to joint inflammation and destruction. Production of high affinity autoantibodies in RA suggests presence of these specialized lymphoid structures, GCs, which are usually found within secondary lymphoid organs, such as the spleen and lymph nodes, but have been observed in ectopic locations like inflamed joint tissues as well [163, 164]. Ectopic GCs were reported to be present in 25–50% of RA patients. However, it was observed that ectopic GCs might not be the major contributors of autoantibodies during inflammatory responses in RA patients [165]. Hence, contribution of GCs to clinical RA is far from clear. In collagen-induced arthritis (CIA), a classical experimental model of RA, GCs are present in both the limbs and secondary lymphoid tissues [166]. Upon collagen immunization, GCs were formed [167] and found to be indispensable for the development of CIA [168].

Analysis of mutations in genes encoding the immunoglobulin-variable (IgV) region in various autoimmune diseases showed that autoimmune B cells contain more IgV mutations than healthy B cells [169]. However, IgV mutations in RA GC B cells are directed only against selected few antigens [170]. Interestingly, studies with a germline-encoded anti-CII IgH replacement mouse strain revealed that self-antigen-specific B cells were neither deleted nor anergized. IgH/L chain sequence data of B cell clones generated from these mice revealed lack of somatic mutations in the autoreactive B cells, but the monoclonal antibodies generated from these mice induced arthritis when combined with another arthritogenic antibody, which suggests pathogenic potential of germline-encoded autoantibodies [171]. It was reported earlier that different genetic regions and their epistatic interactions control autoantibody synthesis [172, 173] and CII epitope-specific antibody response is controlled by IgV(H) gene polymorphisms [174].

Thus, autoantibodies produced either directly or as constituents of immune complexes can trigger inflammation [175, 176]. Passive transfer of purified IgG antibodies from RA patients in naive [177] or mice deficient in the low-affinity inhibitory Fc receptor, Fc*γ*RIIB [178], induced arthritis. Arthritis induced by the passive transfer of antibodies by binding to its target antigens involves Fc*γ*R-bearing granulocytes, mainly neutrophils and macrophages, and complement activation but without the help of adaptive immune responses [179]. Apart from the inflammation-dependent mechanisms, antibodies could also be directly pathogenic to the cartilage independent of inflammatory cells and factors [180]. Certain anti-CII monoclonal antibodies impaired cartilage formation [181], inhibited collagen fibrillogenesis [182], and disassembled CII fibrils in the extracellular matrix with or without increased matrix synthesis [181], possibly compromising the integrity of the cartilage matrix. Interestingly, anti-CII mAbs induced pain-like behavior that was observed prior and after the appearance of clinical symptoms of arthritis, with the involvement of spinal glia [183], and the cartilage binding antibodies were shown to induce pain through immune complex-mediated activation of neurons [184].

**Figure 3:**
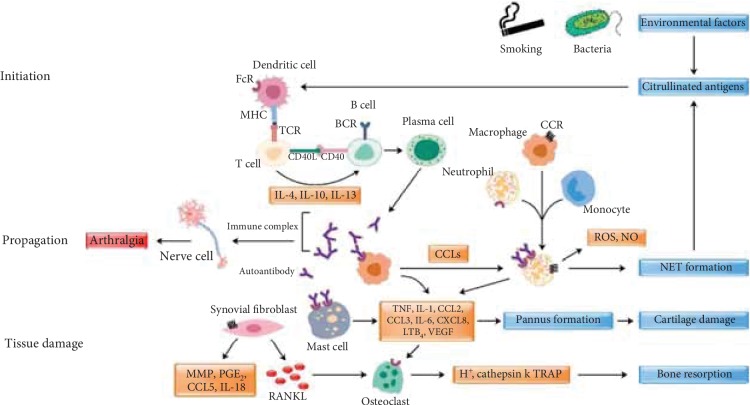
Rheumatoid arthritis pathophysiology. This figure describes different immune cells participating in the initiation, propagation, and tissue damage stages of RA. Environmental factors may trigger generation of posttranslationally modified autoantigens (neo-epitopes) that can be presented by professional antigen-presenting cells to T cells in the context of arthritis-susceptible genetic background. The activation of T cells leads to increased T-B cell cooperation, secretion of cytokines, differentiation of B cells to plasma cells, and production of autoantibodies. These autoantibodies can induce pain as well as inflammation-dependent and inflammation-independent downstream effector mechanisms leading to activation of cells, secretion of proinflammatory cytokines and proteases, which can destroy the cartilage and bone. During this process, neutrophils form NETs, a source for more citrullinated antigens, completing a vicious cycle that can propagate joint inflammation further. CD40: cluster of differentiation 40/co-stimulatory protein present on antigen-presenting cell (APC); CD40L: cluster of differentiation 41-ligand present on Th cell, bind to CD40 to activate APC; TCR: T cell receptor; BCR: B cell receptor; MHC: major histocompatibility complex/surface receptor, with its ligand-activated TCR; FCR: FC receptor/protein receptor present on immune cells; CCR: c-c-motif receptor/beta chemokine receptor; CCL5: chemokine [c-c-motif] ligand-5/RANTES/its chemotactic for neutrophils; CCL2: chemokine [c-c-motif] ligand-2/MCP1; CCL3: chemokine [c-c-motif] ligand-3/MIP-1; CXCL8: IL-8; LTB: TNF-C/induce inflammatory response; VEGF: vascular endothelial growth factor; pannus: abnormal fibrovascular tissue lies over joint surface; MMP: matrix metalloproteinase; PGE: prostaglandin E; IL-1B: interleukin-1-beta; cathepsin k: enzyme controlling bone remodeling; ROS: reactive oxygen species; NO: nitric oxide; IL-4: interleukin-4; IL-10: interleukin-10; IL-13: interleukin-13; T cell: T lymphocyte; B cell: B lymphocyte; TNF: tumor necrosis factor; IL-1: interleukin-1 [192].

In RA, ACPA is associated with arthralgia before the onset of inflammation and a more aggressive disease ensues, suggesting potential pathogenic effects of the ACPA response [185]. Binding of ACPAs to osteoclasts released IL-8, leading to bone erosion [186] and also enhanced osteoclast differentiation from monocyte-derived or circulating CD1c^+^ DCs by increasing the release of IL-8 [187]. Upon binding to its target antigens, ACPAs also induced joint pain by activating sensory neurons via CXCL1/IL-8, released from CD68^+^ osteoclasts in an autoantibody-dependent manner, and blocking the chemokine receptors for CXCL1/2 attenuated ACPA-induced hypersensitivity [188]. Furthermore, ACPAs induced macrophages to secrete TNF-*α*-mediated activation of complement cascades [189] and Fc*γ*RIIa-dependent activation of platelets [190]. High titers of RF are also associated with joint erosion and extra-articular manifestations, leading to poor prognosis [191]. Moreover, synovial mast cells express Fc*γ*RIIA and can be activated by IgG-ACPA and Toll-like receptor (TLR) ligands, and the combined activation of mast cells via these pathways greatly enhances inflammation in the synovial tissue of RA patients [191]. Possible pathophysiological mechanisms involved in RA are depicted in Figure 3, and the autoantibodies occupy a central part in them.

## 9. IgG Glycosylation

Importance of posttranslational protein modifications in the rheumatological diseases has been reviewed earlier [193]. In this context, the role of autoantibody glycosylation in the development of arthritis has been widely reported. Antibodies are composed of 82–96% of protein and 4–18% of carbohydrates [194]. In IgG, N-linked glycans are present both at asparagine 297 on the CH2 part of the Fc domain and in 10–20% of the Fab part [195–197]. In the Fc part, N-acetyl glucosamine, mannose residues with extensions of galactose, sialic acid, fucose, and bisecting N-acetyl glucosamine are present asymmetrically in both the constant CH2 domains, whereas Fab glycosylation is present in the complementarity determining regions of both heavy and light chains and framework regions [198]. IgG glycosylation is associated with inflammation and affects most of the antibody-mediated effector functions [199, 200], which are dependent on the activation of Fc*γ*Rs and complement, and is regulated by Fc glycans. After binding to Fc*γ*Rs or complement, antibodies induce cellular cytotoxicity and cellular phagocytosis as well as cytokine secretion or complement-dependent cytotoxicity, respectively. Decreased galactosylation and sialylation of serum IgG is associated with RA patients [201–206] and in animal models [207]. In RA patients, levels of IgG galactosylation, bisection, and fucosylation are altered [201, 204, 208–212]. In addition, defective galactosylation in the IgG-Fc glycans was observed in RA patients [204, 213, 214] and arthritic MRL-lpr/lpr mice [215]. It was found that agalactosyl IgG has significantly reduced binding to Clq and to Fc*γ*Rs [216]. Clustered IgG in the synovial cavity facilitates multiple presentation of G0 glycans to mannose-binding protein that can lead to complement activation [210], and the mannose-binding lectin activity and G0 glycans correlate with arthritis onset [217]. Treatment of RA patients also changes the profile of IgG glycosylation [218, 219]. Similarly, agalactosyl IgG is associated with disease activity in experimental arthritis [220]. In addition, increased Fab glycosylation was also reported from RA patients [221, 222].

In mice, inhibiting sialylation in activated B cells increased joint inflammation, whereas sialylation of anti-CII monoclonal antibodies (mAbs) attenuated their pathogenic potential [223]. Similarly, pathogenic properties of KBN sera were altered when sialic acids attached to IgG-Fc were cleaved using sialidase or after injection of sialic acid enriched IgG-Fc fragments [224]. Significant changes in IgG-Fc galactosylation and fucosylation in ACPA prior to the onset of RA were observed [205], which is also dependent on IgG subclass [225]. Furthermore, desialylated immune complexes enhanced osteoclastogenesis and mice treated with the sialic acid precursor N-acetylmannosamine increased IgG sialylation leading to decreased bone loss [226]. Moreover, increased Fab glycosylation of ACPA modulated their binding to citrullinated antigens [227].

These studies clearly demonstrate that appropriate modification of the Fc glycosylation status of antibodies could very well attenuate the effector phase of arthritis. In this direction, a bacterial enzyme that can cleave the IgG-Fc sugar molecules specifically was tested. EndoS is a secreted endoglycosidase enzyme from the Gram-positive bacteria *Streptococcus pyogenes* (Group A streptococcus), which specifically hydrolyzes the conserved *β*-1,4-di-*N*-acetylchitobiose core of the IgG-Fc [228], and it is highly specific for human IgG [229]. Removal of the Fc glycan with EndoS causes the Fc domains to deform, leading to diminished binding to Fc*γ* receptors [230] and complement activation. EndoS treatment abrogated arthritogenicity of anti-CII mAbs [231]. Furthermore, EndoS treatment disturbed the formation of stable and larger immune complexes on the articular cartilage surface by cleaving specific sugars present on IgG-Fc, which led to attenuation of joint inflammation [232]. These studies open up a new strategy for specifically modifying the IgG-Fc sugars for the treatment of arthritis patients in the future.

## 10. Conclusion

RF and ACPA are the two most iconic autoantibodies in diagnosis facilitating treatment and prognosis of RA. RF and ACPA have similar diagnostic values. However, ACPAs are certainly more specific than IgM-RF, but in most studies, IgM-RF has been shown to be more sensitive than ACPA. Moreover, IgA-RF as well as IgG-RF are more specific than the IgM isotype alone. However, because of the higher sensitivity and specificity, ACPA has more diagnostic value than RF. Detection of both RF and ACPA can predict the extent of joint damage, and RF is also associated with extra-articular lesions. Moreover, differential alterations in the levels of these two autoantibodies during treatment reflect different underlying mechanisms operating during RA. High affinity autoantibodies are synthesized by plasma cells present in the specialized lymphoid structures (GCs) that are present in secondary lymphoid organs as well as in the inflamed joint tissues. These autoantibodies can induce cartilage damage both in an inflammation-dependent and an inflammation-independent manner. Variations in IgG-Fc glycosylation affect autoantibody effector functions and subsequent inflammation that are dependent on binding to the effector components (Fc*γ*R and complement) of the end-stage effector phase of arthritis. Hence, modification of IgG-Fc N-glycans by glycoengineering or by using specific glycolytic enzymes could be a useful strategy for the treatment of several IgG-dependent autoimmune pathologies.

## Figures and Tables

**Table 1:** List of autoantibodies to citrullinated antigens detected in RA patients.

Antigen	Continent	Country	Sequence	Prevalence	Reference
CCP	Europe	Sweden	Not provided	49-57.4%	[89, 90]
		Italy		28.7-72%	[61, 90]
		UK		10%	[91]
		Denmark		44.9%	[92]
		Netherlands		50-75%	[90, 93, 94]
		France		88.9%	[95]
		Belgium		66.1%	[51]
		Austria		81%	[96]
		Poland		66.7%	[97]
		Spain		36.3-66.3%	[90, 98–100]
		Rome		61-70%	[101, 102]
	North America	USA		61.3-85%	[103–105]
		Canada		48.8%	[106]
	South America	Brazil		84.8%	[107]
	Asia	China		67.9-71.3%	[108, 109]
		Bangladesh		92.3%	[110]
		Japan		75-87.9%	[111–113]
		Thailand		60.3%	[114]
		Israel		80%	[115]
		Sri Lanka		71%	[116]
	Africa	Africa		87%	[117]

CCP-2	Europe	Sweden	Not provided	59-81%	[118–121]
		Netherlands		54-82%	[84, 94, 122–125]
		Hungary		75%	[126]
		Italy		88%	[127]
		Spain		64.2%	[79]
	Asia	Malaysia		64%	[118]
	Africa	Africa		82%	[35]

CCP-3	Africa	Africa	Not provided	77%	[35]
	Europe	Hungary	Not provided	79%	[126]

CCP-3.1	Europe	Hungary	Not provided	84%	[126]

Collagen II	Europe	Sweden	Not provided	Cit-CII_355-378_ (13%)	[118]
				Cit^360,365^-CII_359-369_ (33.5%)	[121]
	Asia	Malaysia	Not provided	Cit-CII_355-378_ (17%)	[118]

Vimentin	Europe	Sweden	VYAT-Cit-SSAV-Cit-L-Cit-SSVP	Cit^64,69,71^-vimentin_60-75_ (29.8-44%)	[27, 28, 118, 128]
			Not provided	Cit-vimentin_2-17_ (11.7-32%)	[118, 121]
			Not provided	Cit-vimentin_1-16_ (2.7%)	[119]
			VYAT-Cit-SSAV-Cit-L-Cit-SSVP	Cit^63,68,70^-vimentin_59-74_ (38%)	[119, 129]
		Poland	Not provided	58.8%	[97]
		Netherlands	VYAT-Cit-SSAV-Cit-L-Cit-SSVP	Cit^63,68,70^-vimentin_59-74_ (58%)	[123]
	Asia	Malaysia	VYAT-Cit-SSAV-Cit-L-Cit-SSVP	Cit^64,69,71^-vimentin_60-75_ (54%)	[27, 28, 118, 128]
			Not provided	Cit-vimentin_2-17_ (25%)	[118]

Fibrinogen-alpha	Europe	Sweden	HHPGIAEFPS-Cit-GKSSSYSKQF	Cit^573^-fibrinogen-*α*_563-583_ (21.9-43%)	[118, 130]
			Not provided	Cit-fibrinogen-*α*_27-43_ (19%)	[119]
			AEGGGV-Cit-GPRVVE	Cit^35^-fibrinogen-*α*_29-41_ (20.2%)	[131]
			KDLLPS-Cit-D-Cit-QHLPLIK	Cit^216,218^-fibrinogen-*α*_201-225_ (12.7%)	[131]
			QMRMELE-Cit-PGGNEIT-Cit-GGSTSYG	Cit^263,271^-fibrinogen-*α*_256-278_ (21.0%)	[131]
			NVSPGT-Cit-Cit-EYHTEK	Cit^425,426^-fibrinogen-*α*_419-432_ (17.0%)	[131]
			SKQFTSSTSYN-Cit-GDSTFESKS	Cit^591^-fibrinogen-*α*_580-600_ (9.8-14.4%)	[118, 121, 130]
		Netherlands	Not provided	Cit-fibrinogen-*α*_27-43_ (28%)	[123]
	Asia	Malaysia	HHPGIAEFPS-Cit-GKSSSYSKQF	Cit^573^-fibrinogen-*α*_563-583_ (39%)	[118, 130]
			SKQFTSSTSYN-Cit-GDSTFESKS	Cit^591^-fibrinogen-*α*_580-600_ (19%)	[118, 130]
	Africa	Africa	GP-Cit-VVE-Cit-HQSACKDS	Cit^38,42^-fibrinogen-*α*_36-50_ (45%)	[132, 133]

Fibrinogen-beta	Europe	Sweden	NEEGFFSA-Cit-GHRPLDKK	Cit^44^-fibrinogen-*β*_36-52_ (42-64.9%)	[118, 129]
			APPPISGGGY-Cit-ARPAKAAAT	Cit^72^-fibrinogen-*β*_62-81_ (19-20%)	[118, 130]
			APPPISGGGYRA-Cit-PAKAAAT	Cit^74^-Fibrinogen-*β*_62-81_ (25.2-30%)	[118, 130]
			APPPISGGGYRA-Cit-PAKAAAT	Cit^74^-fibrinogen-*β*_62-81a&b_ (14.9-35.6%)	[121]
		Netherlands	NEEGFFSA-Cit-GHRPLDKK	Cit^44^-fibrinogen-*β*_36-52_ (60%)	[123]
	Asia	Malaysia	NEEGFFSA-Cit-GHRPLDKK	Cit^44^-fibrinogen-*β*_36-52_ (47%)	[118, 129]
			APPPISGGGY-Cit-ARPAKAAAT	Cit^72^-fibrinogen-*β*_62-81_ (18%)	[118, 130]
			APPPISGGGYRA-Cit-PAKAAAT	Cit^74^-fibrinogen-*β*_62-81_ (15%)	[118, 130]
	Africa	Africa	Cit-PAPPPISGGGY-Cit-A-Cit	Cit^60,72,74^-fibrinogen-*β*_60-74_ (73%)	[132, 133]

Filaggrin	Europe	Sweden	HQEST-Cit-G-Cit-SRGRSGRSGS	Cit^312,314^-filaggrin_307-324_ (44-46.8%)	[118, 121]
		Poland	HQEST-Cit-G-Cit-SRGRSGRSGS	23.5%	[97]
	Asia	Malaysia	HQEST-Cit-G-Cit-SRGRSGRSGS	Cit^312,314^-filaggrin_307-324_ (42%)	[118]

Enolase-alpha	Europe	Sweden	KIHA-Cit-EIFDS-Cit-GNPTVE	Cit^9,15^-enolase_5-21_(40-50%)	[29, 118, 134]
			KIHA-Cit-EIFDS-Cit-GNPTV	Cit^9,15^-enolase_5-20_ (25%)	[119]
			KIHA-Cit-EIFDS-Cit-GNPTVE	Cit^10,16^-enolase_5-21_ 68.1%	[121]
		Poland	KIHA-Cit-EIFDS-Cit-GNPTVE	70.6%	[97]
		Netherlands	KIHA-Cit-EIFDS-Cit-GNPTV	Cit^9,15^-enolase_5-20_ (32%)	[123]
	Asia	Malaysia	KIHA-Cit-EIFDS-Cit-GNPTVE	Cit^9,15^-enolase_5-21_(23%)	[29, 118, 134]
	Africa	Africa	KIHA-Cit-EIFDS-Cit-GNPTVE	Cit^9,15^-enolase_5-21_ (72%)	[117]

**Table 2:** List of autoantibodies to unmodified antigens detected in RA patients.

Type	Continent	Country	Antigen	Positive rate	Reference
RF	Europe	UK	IgG-Fc	13-72%	[91, 135]
		Netherlands		56.9-67%	[81, 90, 136]
		Sweden		55-67.8%	[89, 120]
		Europe		75%	[137]
		France		80.2%	[138]
		Poland		68.6%	[97]
		Italy		41.3-95%	[61, 90, 127, 139, 140]
		Spain		43.2-67.5%	[90, 98, 99]
	North America	USA		62.1-77%	[103, 104]
		Canada		57.7%	[106]
	South America	Brazil		63%	[107]
	Asia	China		71.4-76.9%	[108, 109]
		Sri Lanka		69%	[116]
		Iran		82.7%	[141]
		Taiwan		66.7%	[142]
		Japan		68.1-87.9%	[111, 113, 143–145]
		Bangladesh		94.23%	[110]
		Thailand		73.1%	[114]
cRF (IgA, IgG, IgM)	Africa	Africa		87%	[117]
IgM-RF	Europe	Denmark		55.6%	[92]
		Netherlands		21-75%	[84, 93, 94, 122–125]
		France		83.3%	[95]
		Hungary		70.6%	[126]
		Italy		76.5%	[61]
		Rome		65.3-68%	[101, 102]
		Austria		78.6%	[96]
		Belgium		88.7%	[51]
		Spain		60%	[79, 100]
		Sweden		59%	[94]
	Asia	Israel		80%	[115]
		Japan		86%	[112]
	South America	Chile		90%	[146]
IgG RF	Europe	Italy		59.1%	[61]
		Austria		78.6%	[96]
		Netherlands		24%	[122]
IgA-RF	Europe	Italy		61.4%	[61]
		Austria		73.8%	[96]
		Netherlands		33-60%	[122, 123]

Anti-GPI antibodies	Europe	France	GPI	28.4-45·4%	[76]
	North America	USA		15-49%	[77, 147]
	Asia	Japan		12-18.5%	[113, 143]
		China		75.0%	[109]

Anti-CarP antibodies	Europe	Netherlands	CarP	10-49.2%	[81, 84, 90, 94, 123–125]
		Sweden		26-42.2%	[79, 90, 94, 98–100, 121]
		Poland		29.4%	[97]
		Rome		34.4-38%	[101, 102]
		Italy		10%	[90]
	North America	USA		47%	[90]
		Canada		38.2%	[148]
	Asia	India		41.5%	[148]

Antinuclear antibody	Europe	Denmark	Nuclear antigens	19.4%	[92]
		France		44.4%	[95]

Antikeratin antibodies	Asia	China	Keratin	48.2%	[109]

Anti-hnRNP/RA33	Europe	Poland	hnRNP/RA33	37.3%	[97]
	Asia	China		7.3-44.7%	[149]
